# Visuo-auditory interactions in the primary visual cortex of the behaving monkey: Electrophysiological evidence

**DOI:** 10.1186/1471-2202-9-79

**Published:** 2008-08-12

**Authors:** Ye Wang, Simona Celebrini, Yves Trotter, Pascal Barone

**Affiliations:** 1 Centre de Recherche Cerveau & Cognition, UMR CNRS 5549, Faculté de Médecine de Rangueil, 31062 Toulouse Cedex 9, France; 2 Department of Neurobiology and Anatomy, University of Texas-Houston Medical School. Houston TX, USA

## Abstract

**Background:**

Visual, tactile and auditory information is processed from the periphery to the cortical level through separate channels that target primary sensory cortices, from which it is further distributed to functionally specialized areas. Multisensory integration is classically assigned to higher hierarchical cortical areas, but there is growing electrophysiological evidence in man and monkey of multimodal interactions in areas thought to be unimodal, interactions that can occur at very short latencies. Such fast timing of multisensory interactions rules out the possibility of an origin in the polymodal areas mediated through back projections, but is rather in favor of heteromodal connections such as the direct projections observed in the monkey, from auditory areas (including the primary auditory cortex AI) directly to the primary visual cortex V1. Based on the existence of such AI to V1 projections, we looked for modulation of neuronal visual responses in V1 by an auditory stimulus in the awake behaving monkey.

**Results:**

Behavioral or electrophysiological data were obtained from two behaving monkeys. One monkey was trained to maintain a passive central fixation while a peripheral visual (V) or visuo-auditory (AV) stimulus was presented. From a population of 45 V1 neurons, there was no difference in the mean latencies or strength of visual responses when comparing V and AV conditions. In a second active task, the monkey was required to orient his gaze toward the visual or visuo-auditory stimulus. From a population of 49 cells recorded during this saccadic task, we observed a significant reduction in response latencies in the visuo-auditory condition compared to the visual condition (mean 61.0 vs. 64.5 ms) only when the visual stimulus was at midlevel contrast. No effect was observed at high contrast.

**Conclusion:**

Our data show that single neurons from a primary sensory cortex such as V1 can integrate sensory information of a different modality, a result that argues against a strict hierarchical model of multisensory integration. Multisensory interaction in V1 is, in our experiment, expressed by a significant reduction in visual response latencies specifically in suboptimal conditions and depending on the task demand. This suggests that neuronal mechanisms of multisensory integration are specific and adapted to the perceptual features of behavior.

## Background

The classical view of multisensory integration, based on anatomical grounds [1], proposes that each sensory modality is processed through separate channels from the sensory receptors to the primary sensory areas and then further integrated into associative unimodal areas converging at the level of cognitive polymodal areas [2]. Indeed, in primates, neuronal responses to more than one sensory modality have been described in areas higher-up in the hierarchy like the frontal, temporal and parietal lobes [3-9]. While these polysensory areas are the best candidates to support sensory fusion, recent studies in humans have surprisingly revealed that multisensory interactions can take place in early stages of sensory processing, in regions thought to be involved in only one modality [10,11]. This result has led to a reappraisal of the cortical regions involved in multisensory integration [12]. In humans for example, imaging [13-16] and EEG studies [17,18] have clearly shown heteromodal responses in sensory areas even at the level of the primary sensory fields. Furthermore, the discovery of heteromodal connections directly linking areas involved in different sensory modalities could be the anatomical support of such interactions [19-21]. For example, in the monkey, the core of the auditory cortex receives direct inputs from both somatosensory and visual areas [19]. It can be inferred that these cortical heteromodal connections, as well as the thalamo-cortical loop [22,23], could be the anatomical pathway responsible for the visual [24-26], somatosensory [27,28] or proprioceptive [29] influences observed in the monkey auditory cortex [30].

In the normal adult cat, some early electrophysiological studies have reported auditory responses in visual areas [31-33], a result which is still controversial [34]. Multisensory integration in the primary visual cortex (V1) of the monkey has not been established, apart from a clear influence of a non visual eye position signal on visual activity [35,36]. However, auditory or visuo-auditory responses in area V1 are highly probable since we have demonstrated direct projections from the auditory cortex (including A1) and the polymodal area STP to area V1 in the calcarine sulcus [21]. Furthermore, the auditory system is activated more precociously that the visual one, and for example the latencies of auditory responses recorded in areas AI and STP are about 35 and 45 ms respectively [37,38]. Consequently it conceivable that an auditory stimulus can modulate the visual responses in V1 where the mean onset latencies are longer, between 50–70 ms [39,40]. As some authors have reported even shorter latencies in V1 when using high contrast stimuli [41], one could expect that an auditory stimulus would affect mostly late visual responses such as the one obtained using non-optimal stimuli (ie. low visual contrast).

We thus conducted an electrophysiological study to look for visuo-auditory interactions at the single cell level in primary visual cortex. Because the auditory projections to V1 are more dense at the representation of the peripheral visual field, a region of space that encompasses most of the auditory receptive field in AI [42,43], our electrophysiological recordings targetted visual cells with RF located between 10 and 20° of retinal eccentricity.

## Methods

The present study is based on data obtained from two monkeys (Macaca mulatta) trained to performed a visual or visuo-auditory oculomotor task. A detailed description of the general methods used in the electrophysiological recording has been reported in a previous study [44]. All experimental protocols, including care, surgery, and training of animals, were performed according to the Public Health Service policy on the use of laboratory animals and complied with guidelines of the European Ethics Committee on Use and Care of Animals.

### Behavioral task

The core of the present study concerns two monkeys (Mk1 and Mk2) trained to perform a visually guided saccadic task during which the visual target could be accompanied by an auditory stimulus (V/*VA active task*). A trial was initiated by the appearance of a fixation point (FP) located at the center of the video screen and of a size of 0.2 degree. The monkey had to direct its gaze and to maintain fixation at this central point. The duration of presentation of the FP was randomized between trials and lasted between 1500 to 1800 ms. Simultaneously, with the extinction of the FP, a peripheral visual target was flashed for 50 ms. The monkey was required to perform a saccade in the direction to the locus of the visual target within 250 ms of its appearance. Responses were considered as correct when the saccades were performed within a window of 4 × 4 degrees centered on the visual target, and in these cases a few drops of fruit juice were delivered to the monkeys as a reward. In half of the trials, presented randomly, a 25 ms sound (a white noise) was delivered from a speaker located at the same eccentricity on the azimuth as the visual stimulus. In such visuo-auditory trials (VA), the visual and the auditory stimuli were presented at the exactly same time. In both conditions (V and VA) the monkey was required to perform a saccade directed toward the visual target and consequently, the auditory stimulus had no behavioral meaning for the animal. Note that we did not train the monkeys to perform a saccade toward the auditory stimulus alone.

The first monkey engaged in the present study (Mk1) was first trained to perform two control tasks before the *V/VA active task*. In a first stage, the monkey was trained to perform a simple passive fixation task (*V/VA passive task*). Following the presentation of the FP (of variable duration from 1500 to 1800 ms), a visual or visuo-auditory stimulus was presented for 500 ms together with the FP. To get rewarded, the monkey had to maintain its fixation until the FP was extinguished.

Further, Mk1 was trained in a visual control task (*V-only control task*), during which the color of the FP informed the animal whether he had to maintain a central fixation (blue FP) or to make a saccade toward a visual peripheral stimulus (Red FP). In this case the visual stimulus was never accompanied by an auditory stimulus. The timing of stimulus presentation was identical to that described for the active task (50 ms).

The monkey Mk1 was engaged successively in each of these different protocols for several months, a period during which electrophysiological recordings were performed in the primary visual cortex (see below). Mk2 was trained from the beginning to do the VA active task.

### Apparatus and electrophysiological recording

**Figure 1:**
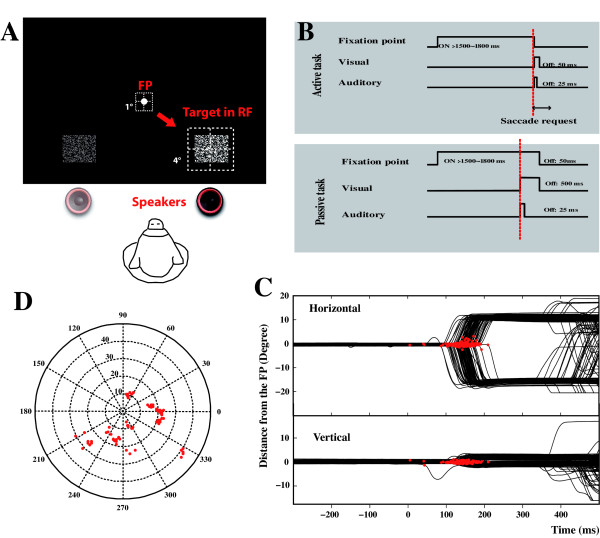
**Experimental protocol.A**. In the active Visuo-Auditory task, the monkey had to perform a saccade toward a visual cue located on the right or left side of the central fixation point. The visual stimulus was placed inside the neurons RF or at the opposite location. Auditory speakers were placed just below the visual location. Dashed squares represent the control windows regulating the eye movement for both the fixation and the saccadic periods. **B**. Temporal succession of the visual and auditory events in the active (upper panel) or passive (lower) tasks. **C**. EEG recordings of a monkey (Mk1) during a saccadic task oriented toward a visual cue located at 16 degrees on the left or right site of the fixation point. The upper and lower panels correspond to the horizontal or vertical eye movements respectively. Red dots represent the saccade latencies as described in the method section. Note that the anticipatory saccades obtained at latencies lower that 30 ms were not included into the analysis. **D**. Polar plot showing the receptive fields location of the recorded single cells. Most of the cells have RFs located at retinal eccentricities beyond 10°.

The visual stimuli, delivered by a Vision Research Graphics system with a refresh rate of 120 Hz, were presented in total darkness on a video screen located 50 cm in front of the monkey. The FP consisted of a single dot of 0.2° of size. Peripheral visual stimuli consisted of dynamic random dots (dRD, density 20%, and dot size 3.5 min of arc) presented at either side of the central FP in a random order. Two or three contrast levels of the dRD were used, from low (15%) to medium (55%) or high values (88%). During the behavioral sessions, the visual stimuli were 5° of size and presented at 10 or 20° of retinal eccentricity in azimuth, and -5 or -10° in elevation. During the electrophysiological recording sessions, the size and the location of the dRD were adjusted to the size and location of the cell receptive fields. Auditory stimuli (white noise, 72 dB SPL) were presented through a multi-channel sound card to one of 6 speakers located just below the video screen (at about 3 degrees below the lower border of the monitor). The horizontal position of the speakers was adjustable to be spatially adjusted in the horizontal plan just below the visual dRD. Thus the auditory stimuli are matching the horizontal location of the cell receptive fields, but not the vertical one (see figure 1). Further, to minimize the reflection of the sound, the animal was placed in a restricted space (1 by 1 meter for each sides) covered of thick black curtains. Acoustical reflection could be problematic for sound localization. However in our protocol the sound had no meaning to perform correctly the oculomotor task of the monkey and we believe that any sound reflection did not affect the monkey performance. Further, based on previous electrophysiological data obtained in the auditory pathway [45], in situation of reverberent environment, a cell's response to the leading source is always stronger than that observed to the lagging one, especially at short lag/lead delays [46]. In consequence, in absence of a sound-attenuated chamber, we think that in our experimental set-up the main auditory effect observed on V1 cells will be produced by the leading direct sound source located in front of the animal.

Protocols control and data acquisition were executed under REX system. To avoid a jittering when generating the auditory stimulus through Windows system, all stimuli were pre-generated and stored in memory. Then we added a buffer silent time before each auditory stimulus. Triggers corresponding to the beginning of auditory stimuli (from the buffer) and the first visual frame (through VRG) were sent to REX system. The audio buffer length was adjusted to synchronize the visual and auditory stimuli at expected delay.

Aseptic surgery was performed to attach a head-post to the skull and to implant a scleral search coil in both eyes. Single-unit recordings were made in one of the two monkeys (Mk1). Once the monkey had reached a high level of performance, a second surgery was performed to implant a recording chamber above the peripheral visual field representation in V1 located in the calcarine sulcus [47]. The skull was removed within the chamber, and a fixed grid was placed, so that the electrode penetrations were spaced 1 mm apart. Guide tubes were used to help to penetrate the dura. Sterile, tungsten-in-glass electrodes of ~1 MΩ impedance were inserted with a hydraulic microdrive fixed to the recording chamber, perpendicular to the cortical surface. Extracellular recordings were carried out in both hemispheres of the monkey from which the visual responses were previously analyzed for disparity selectivity (see [48]). Action potential waveforms were sorted online with the help of a spike sorting software (AlphaOmega MSD^®^) and only single units recorded through complete trials were selected for analysis.

### Data analysis

The behavioral analysis was derived from the performance of the two monkeys trained to perform the visually and visuo-auditory guided saccadic task (V/*VA active task*). For each trial we determined the saccade latency defined as the first point when the eye position was significantly different from the average eye position signal during the 300 ms prior to stimulus offset. This corresponded to the time at which the difference between the current position and the mean was 2 times greater than the maximum range observed during the fixation period. Then we performed a statistical analysis (Multifactor Anova) to compare the saccade latencies obtained during the V and VA conditions. We used a multi-way ANOVA test to the saccade latency obtained in each monkey. Contrast (3 groups in MK1, 2 groups in MK2), eccentricity (2 groups), and V or VA stimulation (2 groups) were treated as different factors. We checked both single factor and two-factors interactions. In case of p value too low to be computed, as we know it show very high significance, we also indicate F value as references. Post-hoc test was then applied to compare the saccade latencies between individual pair of conditions.

For each neuron in each condition, the neuronal activity was recorded for 20–40 correct trials. Two parameters were studied to analyze the effect of visuo-auditory interactions in V1 cells : the amplitude and the latency of the visual responses. To measure the visual response latency, we first computed histograms of neuronal activity aligned on the stimulus onset. As previously described [48,49], we further smoothed the accumulative line by simulating each spike as a mini gauss function (Amp. = 1; Sigma = 4). So within each 1 ms bin, we got statistical spike numbers of 40 (trial number)*10(gauss summation) ms window. Then we measured the baseline average spike number per bin in the 200 ms prior to stimulus onset, and used it as the Poisson distribution lambda parameter of spontaneous activity. So the threshold of response activity was the smallest number *n *that the Poisson cumulative density function evaluated which equaled or exceeded 0.99. Thus, if the firing property obeyed the same Poisson distribution as the baseline, the spike number within each bin would not exceed this value at 99% confidence. We calculated this number *n *by using the Matlab Poisson function. We then used a detection window of 4 ms and measured activity starting from visual stimulus onset. If the minimum value inside this window was greater than *n*, we determined the response latency as the first point of the window. Because we could only get one latency value for a group of trials, we used bootstrap methods to compare the activity between conditions : shuffle latencies were calculated from the same number of trials in a sample taken randomly from both conditions. This was performed 4000 times to obtain 2000 randomly grouped pairs, from which we calculated the individual difference within pairs. The bootstrap p value is the ratio of pairs for which differences were no less than the values obtained from the experimental data. At the population level, after normalization of the responses, we used an Anova test to analyze the factor effect on response amplitude or latency, and paired t-tests for post hoc comparison.

## Results

### Visuo-auditory interaction: behavioral evidence

**Figure 2:**
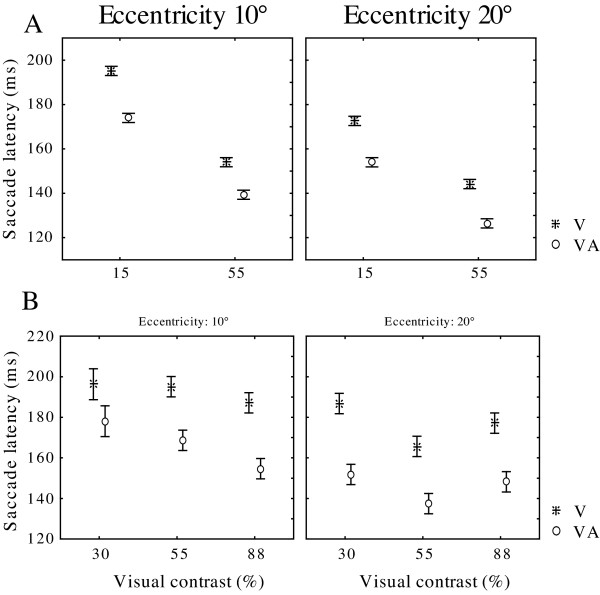
**Saccadic reaction times (± sd) of the individual monkeys** (A: monkey 1; B: Monkey 2) according to the eccentricity of the visual target (10° left panels; 20° right panels) presented at different contrasts and during the visual-only () or visuo-auditory conditions (○). Saccadic latencies are shortened both when increasing the visual contrast and when the visual cue is presented simultaneously with the auditory stimulus (VA conditions).

In a first stage we analyzed the effect of visual conditions on the saccadic reaction times (sRT) performed by the two monkeys (Fig 2). The present data concern behavioral latencies obtained in highly trained monkeys and are based on several thousands of trials. Across the different conditions of stimulation (uni- or bimodal, at different contrasts or eccentricities), sRT values were on average 155,0 ms for Mk1 and 167,6 ms for Mk2 which correspond to the range of values reported in other studies using similar experimental conditions)[50]. First in uni- and bimodal conditions we observed a decrease in sRT in both monkeys when the contrast of the visual cue increased (Anova, Mk1, F = 4012, p = 0; Mk2, F = 20.39, p = 1.64E-9). This was particularly evident for Mk1 during a 20° task in visual-only conditions, with sRT of 172 ms when the contrast was low and of only 144 ms at high contrast. This is in agreement with numerous similar psychophysical studies on RT in both man and monkey [51-53]. Furthermore, in both sensory conditions, the monkeys had much shorter sRT when the saccades were directed toward the more eccentric (20°) peripheral target (Mk1, F = 527.65, Mk2, F = 148.17, p = 0 both cases). On average, independent of the visual contrast, saccade latencies toward eccentric cues located at 20° were 10% shorter than those toward a cue at 10°. This difference in latencies tended to be larger in the visuo-auditory (12.6% shorter at 20°) compared to the visual-only condition (8.6%). Again, this effect of the eccentricity of a target on the saccadic reaction time is similar to that observed in humans [54].

We analyzed next the effect of bimodal stimulation by comparing the sRT in the V-only and VA conditions (Fig 2). As classically reported and resulting from multisensory integration [55], we observed a strong reduction in the saccade latencies in the VA condition compared to the V-only situation. This reduction was observed at all eccentricities (Anova, 10°: Mk1 F = 231.17,, Mk2 F = 66.67, both p = 0; 20° Mk1 F = 404.76, Mk2 F = 271.45, both p = 0) and at all contrasts of the visual target (Anova, Mk1, 15% F = 516, 55% F = 329.7, both p = 0; Mk2 30% F = 107.3, p = 0, 55% F = 69.38, p = 3.33E-16, 88% F = 117.45, p = 0). On average, when combining all conditions, the decrease in sRT ranged from 10% (Mk1) to 15% (Mk2) when saccades were made toward the VA stimulus. The rule of inverse effectiveness [55], proposes that the higher benefits resulting from multisensory integration should be obtained in sensory conditions of low saliency. Thus we searched for an effect of visual contrast on the reduction of sRT during visuo-auditory saccades. In Mk2, for which data were obtained at 3 different contrasts (30–55 and 88%), there was a tendency toward a more pronounced shortening of sRT at low contrast. When saccades were made at 20°, sRT in VA conditions were 19% shorter at a low contrast (187 ms in V-only vs. 152 ms in VA, p = 6.38E-21) while the decrease was only 14% at high contrast (174 ms vs. 150 ms, p = 7.29E-23). However we did not replicate these results in the second monkey or in all conditions. In Mk1, at 20°, the reduction was similar at low (11.1% decrease) and high contrast (11.9% decrease). Thus, we observed a constant decrease in sRT at all the visual contrasts used, data which seem to contradict the rule of inverse effectiveness. However, this could be due to the level of training. When analyzing the data during the first sessions of the behavioral training of Mk1 (not shown), we found a stronger decrease in sRT at low contrast, but at that time the monkey was not performing at an efficient level and his saccade latencies were much longer. This effect disappeared after extensive training over several weeks.

### Visuo-auditory interaction: electrophysiological evidence

The present study is based on three sets of visual responsive single units (total n = 136) recorded in the primary visual area V1 of one monkey (Mk1). Each set of cells was obtained during a single behavioral condition (V/*VA active task n = 49; V/VA passive task n = 45; V-only control task n = 42*), all cells were recorded in peripheral V1 and most of them (69%) were located in the upper bank of the Calcarine sulcus (Fig 1) and present a receptive field located over 10° of eccentricity in the lower visual field. The size of the receptive fields were ranging between 1 and 4° (see [48]) characteristic of those cells recorded in the peripheral representation of V1.

#### Auditory modulation of visual responses in V/VA active task

The visual responses (discharge rate and latency) of 49 isolated V1 neurons were analyzed during the active visual and visuo-auditory tasks (Table 1). In the V-only conditions, the cells showed a strong phasic activity in response to the dRD and both the magnitude (Anova, F = 4.5, p = 0.0135) and latencies (Anova, F = 58.36, p < 0.0001) of the responses were affected by the contrast level as classically reported for V1 cells [56]. When comparing the response rates, we observed that while the discharge rates were similar at high (88%) and medium (55%) contrasts (54.5 spk/s and 52.2 spk/=s respectively, paired t-test p = 0.18, ns), the neuronal activity was, on average, much lower at the low contrast of 15% (33.9 spk/s, paired t-test, p < 0.001, both comparisons). The cell latencies were also sensitive to the contrast level and we observed a progressive increase in the mean latency (table 1, paired t-test p < 0.001 for all comparisons) when presenting stimuli from the high (49.2 ms), medium (64.5 ms) or low contrasts (100.7 ms). In the bimodal condition (VA active task), we observed exactly the same influence of visual contrast on the neuronal responses as expressed by an increase of discharge rate (Anova, F = 3.65 p = 0.0296) and a decrease of latencies when increasing the contrast levels (Anova, F = 52.31 p = 3.33E-16, Table 1). Thus the simultaneous presentation of an auditory stimulus has no effect on the contrast dependent relationships of the visual responses of V1 neurons.

**Table 1:** Response rates and latency values (± se) of V1 single units recorded during the V/VA active tasks using three different contrast levels.

	Response Rate (spk/s)		Latency (ms)	
	
	V-only	AV	V-only	AV
Low level (n = 17)	33.9 ± 5.0	35.7 ± 5.1	100.7 ± 6.2	96.8 ± 6.2
Mid-level (n = 39)	52.2 ± 4.0	52.7 ± 4.1	64.5 ± 2.5	61.0 ± 2.3
High level (n = 45)	54.4 ± 3.9	54.1 ± 3.8	49.2 ± 1.8	49.0 ± 2.0

**Figure 3:**
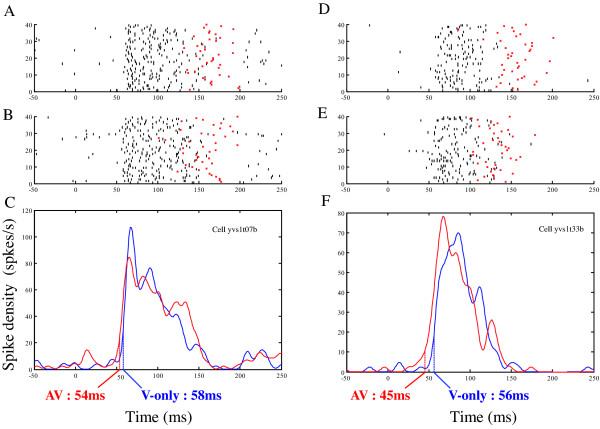
**Examples of activity of two V1 single cells that present a significant reduction of their visual responses latency during the bimodal visuo-auditory conditions (AV).** A and D represent rasters of the cells activity in the visual-only condition, while B and E show the activity of the same cells in the visuo-auditory conditions. The red dots indicate the time at which the monkey is making a saccade toward the visual target. C and F represent the response peristimulus time histogram to visual (blue) or visuo-auditory (red) stimuli. In both cells, the AV response latency is shorter compared to the V-only condition.

**Figure 4:**
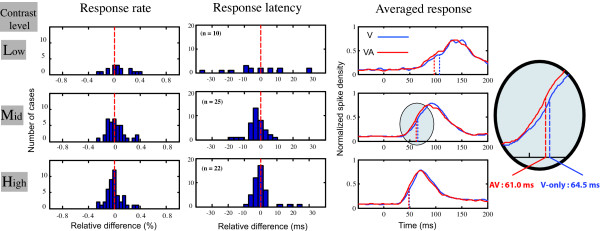
**Effects of a visuo-auditory stimulus on the visual responses obtained from 49 single units recorded in V1 during a visuo-auditory saccadic task performed at three contrast levels (15%, upper row, 55%, middle row and 88%, lower row).** The right panels represent the averaged normalized responses of the cells population during the V-only (blue) or visuo-auditory (red) conditions. The other panels show the distribution of the relative difference in the response rates (left) or latencies (middle) of the cells when the V-only and VA conditions are compared for each individual cell. A value of "0" means no difference, while negative values represent a decrease in the VA compared to V-only condition. At high and mid-contrasts no effects are observed concerning the response rate, while we observed a shift of the distribution of latencies toward negative values, indicating a shortening of the visual latency during the bimodal condition. The numbers of cells showing a reduction in latency are indicated in the brackets.

When we compared the V and AV conditions at constant contrasts, the simultaneous presentation of a spatially congruent auditory stimulus affects the cells activity by a change in the response latency. This is illustrated in two examples in figure 3. Both cells showed a characteristic phasic response to a dRD presented in their RF. While the frequency discharge was similar during the V-only and AV conditions (paired t-tests, both cells, p > 0.05), the two neurons showed a significant decrease in latency. For example, cell #33 (left panel) when stimulated at a midlevel contrast, had a mean latency of 56 ms during the visual task, a value that was reduced to 45 ms in the visuo-auditory conditions (bootstrap, p = 0.0415), while the spike discharge remained constant (35 and 38 spk/s respectively, paired t-tests, p = 0.4874, ns). This general effect of the auditory stimulus on the visual responses held when the analysis was performed at the population level. First when comparing the V-only and VA conditions, we did not see any significant change in the response rate of the cells at high and midlevel contrasts (Table 2, paired t-test p > 0.5 ns for both conditions). However, at low contrasts we observed a slight increase in the response rate from 33.9 spk/s to 35.7 spk/s a difference that is just below the significance level (paired t-test, p = 0.04). The middle panel in fig 4 shows the distribution of the relative differences of response rates (in %) between the uni- and bimodal conditions of all cells at each visual contrast. In the two higher contrast conditions, the distribution is centered at 0, corresponding to an absence of variation of the neuronal discharge between the V and AV presentations.

**Table 2:** Response rates and latency values (± se) of V1 single units recorded during the V/VA passive tasks and the V-only control task using a middle (55%) contrast value.

	Passive task		Visual control task	
	
	V-only	AV	Visual Fixation	Visual Saccade
Response Rate (spk/s)	29.5 ± 2.6	29.8 ± 2.8	28.7 ± 2.3	29.4 2.2
Latency (ms)	65.0 ± 4.7	65.2 ± 4.3	44.0 ± 1.4	43.5 ± 1.5

However, the cell response latency was globally reduced when the auditory stimulus was delivered simultaneously with the visual target (Table 1). At the population level and at each contrast, the cells latency tended to be shorter. This is illustrated in Fig 4 by a leftward shift toward negative values in the distribution of the relative differences (in ms) when comparing the A and VA conditions. This effect reached a statistically significant level only for the 55% middle visual contrast (paired t-test, p = 0.009). In this condition, the mean latency was 64.5 ms in the unimodal visual stimulation against 61.0 ms in the VA condition, corresponding to a global decrease of more than 5%.

At 15% contrast, the VA stimulation lead to similar values of latency compared to the V-only task (96.8 ms vs 100.7 ms respectively), a difference which was not statistically significant (paired t-test, p = 0.43 ns) probably because of a greater variability in the measured latency due to a strong reduction in the cell discharge (see above) when presenting this low contrast visual stimulus.

Finally, at high contrast (88%) the neurons showed very comparable latencies (49.0 in VA vs 49.2 ms in V-only, paired t-test, p = 0.82 ns).

**Figure 5:**
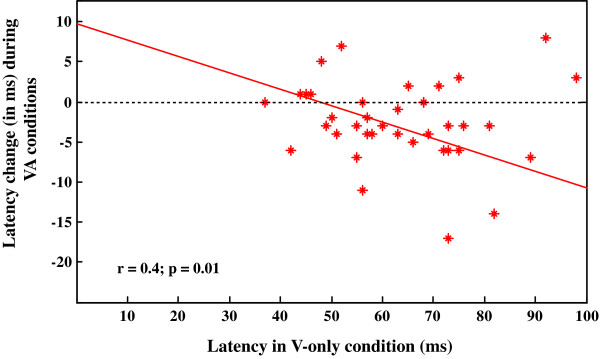
**Relationship between the latencies of the cells in the V-only active task and their respective changes (in ms) when tested in the AV active task.** We observed a statistical inverse relation (P = 0.01; Pearson test) corresponding to a larger reduction for the cells showing longer response latencies.

The decrease in latency at the mid contrast level did not similarly affect all visual cells in V1. A correlation analysis between the absolute latency values in the V-only condition and the relative change (in ms) that occurred during the VA stimulation, revealed an inverse relationship (r = -0.4, p = 0.01, Pearson test, see Figure 5). This means that the cells with the longer latency showed a greater reduction in the bimodal condition, a mechanism that consequently should globally increase the rate of visual processing in area V1. In the other visual conditions (high and low contrasts), the correlation analysis did not reach a statistically significant level (both cases, p > 0.05).

To conclude, we observed that the concomitant presentation of an auditory signal simultaneously with a saccadic visual target induced a reduction of the latency of V1 cells that depended on the contrast of the visual target. Furthermore, the discharge rates of the neurons remained unaffected by bimodal stimulation except during visual conditions that approach the perceptive threshold.

#### Absence of visuo-auditory interactions in the V/VA passive tasks

**Figure 6:**
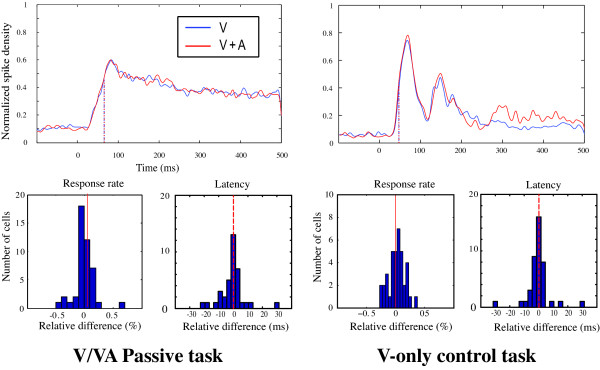
**Left: effects of a visuo-auditory stimulus on the visual responses obtained from 45 V1 single units recorded during a passive visuo-auditory task.** No effects are observed either on the response rate or on the visual latency. Right: absence of an effect of the behavioral paradigm on the activity of 42 V1 neurons during the visual-only condition. When comparing the neuronal activity of the same cells during a visual passive fixation task to a visual saccadic task, we did not observe a modulation of the response rate or response latency. Conventions as in Figure 5.

We analyzed the effect of a visuo-auditory stimulus on the activity of a different set of 45 V1 neurons in a passive task during which the monkey maintained a central fixation while the peripheral stimulus was delivered in the cell's RF. In this case, the results were quite simple in that at all contrasts tested, we did not observe a change in the visual response with the spatially congruent auditory stimulus (Table 2, fig 6). First, the response rate remained unchanged between the V-only and VA conditions (29.5 and 29.8 spk/s respectively, paired t-test, p = 0.69 ns) as reported in the active task. However, in contrast to the effects observed in the saccadic task, the cell visual latencies were the same in the two conditions (65.0 ms in V-only vs. 65.2 ms in VA condition, paired t-test, p = 0.95 ns). These results are presented in Fig 6 as the relative changes in discharge rate and latency values, and the distributions are well centered on zero, indicating no difference between the two conditions.

**Figure 7:**
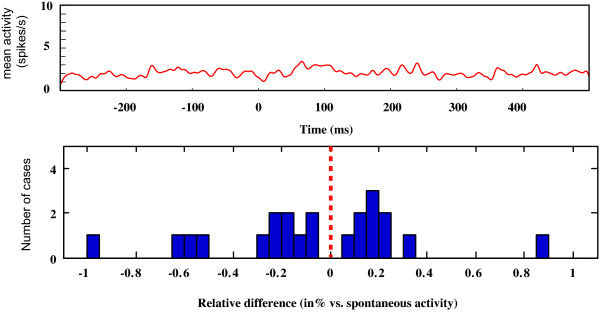
**Average activity of 29 V1 single cells following the passive presentation of an auditory stimulus.** The upper graph shows the average activity of the entire population. No response can be observed following the 25 ms presentation of the broad band noise at time 0. This is also apparent in the lower graph that compares the relative difference of the neuronal activity before and during the auditory presentation. No statistical differences were obtained (see text) confirming the lack of auditory response.

In a subset of neurons (n = 29), we also searched for an effect of the auditory stimulus alone on the neurons activity during a simple central fixation (Fig 7). We did not observe any auditory response. Following a single sound presentation the firing rate of the single cells remained at the same level as the spontaneous activity (paired t-test, p = 0.9056, non significant).

#### Visual responses in the V-only control task

Previous studies in the behaving monkey have shown that neuronal responses in striate and extrastriate cortical areas can be modulated by the behavioral meaning of the stimulus [57,58]. Consequently, we compared the visual responses of a third set of V1 single units (n = 42) during a dual task, a passive central fixation and an active visually guided saccadic task. As explained in the methods, the type of task was indicated to the animal by the color of the fixation point. In this case (Table 2, Fig 6), we did not observe an effect of the task (passive vs. active) either on the frequency discharge (28.7 and 29.4 spk/s respectively, paired t-test, p = 0.25 ns) or on the visual latency (44.0 and 43.5 ms respectively, paired t-test, p = 0.70 ns). This last part suggests that the visuo-auditory interactions that differentially affected the cells in the previous conditions were probably not due to the oculomotor demands of the task in which the monkey was engaged.

## Discussion

The present results demonstrate that in behaving monkeys visuo-auditory interaction can occur at the single cell level at the first cortical stage of processing of visual information, the primary visual cortex V1. Multisensory interactions in V1 are characterized in our experiment by a modulation of V1 responses corresponding to a reduction of the neuronal onset latency. Moreover this effect was dependent on the perceptual charge of the task in which the animal was engaged.

### Visuo-auditory interaction: behavioral evidence

We show that the simultaneous presentation of a sound during a visually guided saccade, induces a reduction of about 10 to 15% in the saccade latency depending on the animal and on the visual stimulus contrast level. Such behavioral improvement resulting from a bimodal visuo-auditory stimulation has been already reported during similar paradigms of spatially oriented behavior in humans [54,59-61], monkeys [50,59], carnivores [62,63] and even in rodents or birds [64,65]. Numerous studies have established the beneficial effect of bimodal stimulation [66] when the experimental sensory conditions respect the rules of spatial and temporal congruencies [55]. In these cases, multisensory integration results in perceptual improvements by reducing ambiguity in various tasks, from simple detections to complex discriminations, memory or learning tasks [67-71]. The decrease in reaction times during a bimodal paradigm has been explained by a co-activation system [72] that violates the race model of independent sensory channels in which the faster modality initiates the motor response. In our study, we did not train the animals to make a saccade toward an isolated auditory cue, so we cannot conclude on the race model. However, we recently reported evidence that such a converging model can account for a shortening in RT in visuo-auditory detection task in the monkey [23].

Multisensory integration is supposed to obey the rule of inverse effectiveness that proposes a higher multisensory benefit when the unisensory stimuli are weak [62,73]. We did not observe such effects and the decrease in sRT was identical when comparing visuo-auditory performances at low or high visual saliencies, a result comparable to that recently reported in a similar behavioral study in the monkey)[50]. We cannot rule out the possibility that if we had used a weaker auditory stimulus it would have produced a change in bimodal gains [23], but it is very likely that this lack of inverse effectiveness is due to the fact that our experiments were performed on highly trained monkeys. It has been shown in monkey, that a continuous training strongly decreases the saccade latency [74], probably reducing the potential range of facilitation induced by the mechanisms of multisensory integration.

### Multisensory interaction at early stages of sensory processing

The delimitation of the polymodal areas associated with multisensory integration was until recently, generally circumscribed to cortical areas in the parietal, frontal and inferotemporal regions of the monkey [5,38,75-77]. However, electrophysiological and functional imaging studies in humans have recently revealed that visual, somatosensory or auditory areas defined originally as unimodal can be the locus of interactions between other non-specific sensory modalities [13,16-18,78-81]. In the monkey, electrophysiological recordings have confirmed that unimodal areas, located at the first stages of the sensory processing hierarchy, can integrate information from a different sensory channel [11].

Until recently, this heteromodal activity had been observed in primates principally in the auditory system. For example, recordings of neuronal activity in the auditory cortex have revealed visual and somatosensory responses in the associative areas of the belt and parabelt [25,27,28,82,83]. In the primary auditory cortex, electrophysiological recordings (current source density) suggest that non-auditory events are of a rather modulatory influence and do not drive activation at the spiking level [84]. For example, proprioceptive information (eye position) can induce changes in the strength of the neuronal discharge in response to a spatially defined sound [29]. Furthermore, it has been proposed that the effect of non-auditory stimuli on AI activity is performed through a modulation of cortical oscillations to allow either enhancement or depression, depending on the timing of the bimodal stimulation [84]. Our results are in agreement with this notion of a modulatory effect and we did not find any auditory response in the single units we tested. The lack of pure auditory response in spite of an auditory modulation of the visual latency suggests that in V1, multisensory interaction could be a subthreshold phenomenon as hypothetized for multisensory interactions in the auditory cortex [24,83]. Because the auditory system is activated faster than the visual one, the auditory stimulus can depolarize the membrane potential of the visual V1 cells, inducing an earlier spiking response compared to the visual-only condition. Such multisensory interaction on cortical sensitivity has been recently suggested by TMS studies in human at both perceptual [85] and behavioral [86] levels.

The main visuo-auditory effect we observed, was a shortening of the visual latencies but only in specific behavioral situations. All together these results suggest that in primates, multisensory integration mechanisms differentially affect sensory responses when they occur in primary or secondary sensory areas [11,24,87-90].

Most of the neuronal rules of interactions between sensory modalities have been established in the Superior colliculus (SC) which is considered to be the key structure for multisensory integration [55]. In the SC, the convergence of different sensory modalities is reflected mainly by an enhancement in neuronal activity in response to a combined multimodal stimulus when spatial and temporal congruencies are respected [91-94]. A modulation (enhancement or depression) of the strength of the unimodal response by bimodal stimulation has been also reported in higher order polymodal areas of the monkey such as the prefrontal, parietal or inferotemporal areas [75,77,95,96] and even in the primary auditory cortex [24,84,97]. However, the proportion of neurons showing enhancement or depression varies strongly across cortical areas. When presenting middle or high contrast visual stimuli, we did not observe such an effect on the response rate in the large sample of visual cells recorded in V1, irrespective of the behavioral paradigm, suggesting that the neuronal mechanisms of multisensory integration are based on rules which are specific to each individual area. However, at low (15%) contrast, the slight increase of the responsiveness of V1 neurons in the AV condition suggests that the rule of inverse effectiveness could apply to V1. We cannot exclude that such effect on the response rate would be more prominent for visual stimuli of even lower perceptive saliency.

In addition, as described in the methods, the visual and auditory stimuli are only spatially congruent in the horizontal azimuth dimension. While the receptive fields of the auditory neurons are large [98] and cover probably the offset that separate the two stimuli, one can speculate that a better spatial congruency between the auditory and visual stimuli would lead to greater effects on V1 cells during bimodal stimulation.

Finally, a rule common to several cerebral loci of multisensory integration is an effect on the response onset latency [75,99]. We observed that in the active task, the visual latency was reduced by about 5%, a result very similar to that reported in the SC [50,99]. This decrease in neuronal response onset, which is in line with a shortening of the visuo-auditory bold response in human V1 assessed by fMRI [13], could participate in the speeding up of the behavioral saccadic responses during bimodal presentation (see below, [62]).

As developed in the introduction, previous anatomical studies have established that sensory fusion was processed through the convergence of the different sensory channels at the level of associative cortical areas [1,2]. The numerous reports of multisensory interactions at low level of sensory processing (present data, [24,27,84]) and acting on early sensory responses, favor a modulatory influence through heteromodal connections linking directly unisensory areas [19-21]. However, such modulatory effect could also originate from non-specific thalamic nuclei that integrate different sensory processing [100]. A cortico-thalamic loop that bypass cortico-cortical connections could thus support fast transmission and provide multisensory and sensory-motor information to unimodal areas [22,101].

### Visuo-auditory interaction: role of the behavioral context

In the alert monkey, we have shown that visual neurons in V1 showed a decrease in response onset when the visual stimuli were presented simultaneously with a sound. However, our main result is that this effect on the visual responses is dependent on the behavioral context : we did not see any changes in V1 neuron latency in a passive situation when the monkey did not perform an oriented saccade toward the spatial location where the auditory stimulus was presented. It could be argued that this difference simply reflects a process of visual spatial attention [102] due to the oculomotor task and not a modulation specifically due to the integration of the auditory stimulus at the neuronal level. In V1 and extra-striate areas, it has been shown that attentional mechanisms [103,104] or behavioral relevance [57,105,106] can affect the characteristics of the neuronal visual response such as the discharge rate, the latency or the neurons selectivity. We did not observe a change in the cell firing rate when comparing neuronal activity in a visual passive and active task without any auditory stimuli. While our comparisons are performed on a different set of neurons, it strongly suggests that the shortening in latency depends specifically on the bimodal conditions in a particular behavioral situation, and not on visual attentional processes linked to the oculomotor demand of the task. However, we are aware that the three paradigms differ in term of attentional loads but in both passive and active AV tasks, the auditory stimulus can involve similar mechanisms of exogenous attention. The distinction between exogenous spatial attention and crossmodal interactions (or integration) is still an open question [107] as both mechanisms result in an improvement in sensory perception [102].

Our results are in complete agreement with studies in humans and animals showing different patterns of multisensory integration according to the behavioral context. First in humans, the detection or discrimination of bimodal objects, as well as the perceptual expertise of subjects, differentially affect both the temporal aspects and the cortical areas at which multisensory interactions occur [18,108]. Similarly the index of multisensory integration computed from the activity of neurons in the deep layers of the Superior Colliculus, is also dependent on the oculomotor behavior of the animal [109]. Finally, while heteromodal visual or somatosensory responses can be obtained in the auditory cortex of a passive or anaesthetized monkey [24,27,87], some authors have reported that some visual responses can be related to task in which the animal is engaged [25].

All together these findings suggests that the neuronal network involved in multisensory integration as well as its expression at the level of the neuronal activity is highly dependent on the perceptual task in which the subject is engaged. Thus multisensory interactions can underly from active perception to attentional mechanisms. This hypothesis is supported by the anatomical pattern of heteromodal connections that directly link areas involved in different modalities. In monkey, such heteromodal connections either link specific sensory representations (retinotopy or somatotopy) of interconnected areas or specific functional regions in each modality [19,21,110].

Such an influence of the perceptual context on the neuronal expression of multisensory interaction has further consequences on the phenomena of cross-modal compensation that occurs after sensory deprivation in animals [111] or humans [112,113]. In blind subjects [114], the efficiency of somatosensory stimulation on the activation of the visual cortex, is maximum during an active discrimination task (Braille reading). This suggests that the mechanisms of multisensory interaction at early stages of sensory processing and the cross-modal compensatory mechanisms are probably mediated through common neuronal pathways.

#### Role of visuo-auditory integration in the primary visual cortex

The effect of an auditory stimulus on V1 responses is probably supported through the direct projections that originate in the auditory (A1 and belt) and multimodal (STP) areas and target V1 [20,21]. As discussed previously [21], the auditory projections to V1 originate mainly from the dorsal auditory stream, specialized in processing spatial information, and reach the peripheral representation of V1. The characteristics of this heteromodal connectivity suggest that this pathway is probably involved in rapidly orienting the gaze toward a sound source located in the peripheral field for which visual acuity is poor. In situations of spatial and temporal congruency, multisensory integration has been shown to facilitate the neuronal responses of neurons of the superior colliculus [115,116], both at the sensory and motor levels [50,59]. Consequently, multisensory integration at the collicular level will allow a direct influence on motor output because the SC is directly involved in the control of oculomotor behavior [117]. A large number of visual areas project directly down to the SC, but in the monkey, the main inputs are originating from the primary visual cortex which constitutes about 20 to 30% of the SC cortical afferents [118]. Consequently the decrease in V1 response latencies during bimodal stimulation can act directly on the response of cells in the SC and speed up the initiation of the saccadic command by the brain stem oculomotor nucleus. However, because the reduction in V1 latencies (5% decrease) does not match the amount of facilitation at the saccadic level (10 to 15% reduction in saccade latency), one should consider other mechanisms outside V1, to transfer the facilitation from the sensory to the motor level.

A remaining question is whether the visuo-auditory interactions reported here at the level of V1 and expressed as a reduction in neuronal latency, represent a *real *multisensory integration or only a sensory combination [119]. In our protocol, auditory and visual stimuli are not redundant signals as the sound has no meaning to perform the task and thus in this way, we should refer to bimodal interactions in V1. However, at the behavioral level, the observed shortening of saccade latency in the bimodal conditions is a phenomenon generally attributed to multisensory integration processing [72]. It is possible that the reduction in latency, especially because it affects mainly the longer ones, will induce a higher temporal coherence of the visual responses across V1. Such a processing has been suggested to increase the cortical synchronization which in turn enhances the speed and reliability of the visual responses [120], and thus could participate to the reduction of RT in bimodal conditions.

## Conclusion

To conclude, our results provide further evidence of the various roles of monkey area V1 in visual perception. Area V1 receives feedback projections from a large number of cortical areas [121]. V1 is connected with areas located at higher levels of the visual processing hierarchy [122,123], with non-visual sensory areas as described above, as well as with the area prostriata [124] which might constitute a gateway to the motor system [125]. This connectivity pattern could be the anatomical support of the neuronal modulation of V1 responses by higher cognitive processes such as attention mechanisms [126,127] or memory tasks [128,129]. The present results suggest that multisensory integration should be added to the list of cognitive processes performed in V1.

## Authors' contributions

YW carried out all experiments and performed statistical analysis. YW, SC, YT and BP participated in the design of the study, the data analysis and wrote the manuscript. All authors read and approved the final manuscript.
